# *OsFRDL1* expressed in nodes is required for distribution of iron to grains in rice

**DOI:** 10.1093/jxb/erw314

**Published:** 2016-08-23

**Authors:** Kengo Yokosho, Naoki Yamaji, Jian Feng Ma

**Affiliations:** Institute of Plant Science and Resources, Okayama University, Chuo 2-20-1, Kurashiki 710-0046, Japan

**Keywords:** Apoplastic Fe, citrate efflux, ^57^Fe distribution, node, rice, transporter.

## Abstract

Iron (Fe) is essential for plant growth and development, but the molecular mechanisms underlying its distribution to different organs are poorly understood. We found that *OsFRDL1* (*FERRIC REDUCTASE DEFECTIVE LIKE 1*), a plasma membrane-localized transporter for citrate, was highly expressed in the upper nodes of rice at the reproductive growth stage. *OsFRDL1* was expressed in most cells of enlarged vascular bundles, diffuse vascular bundles, and the interjacent parenchyma cell bridges of uppermost node I, as well as vascular tissues of the leaf blade, leaf sheath, peduncle, rachis, husk, and stamen. Knockout of *OsFRDL1* decreased pollen viability and grain fertility when grown in a paddy field. Iron was deposited in the parenchyma cell bridges, a few of the cell layers of the parenchyma tissues outside of the bundle sheath of enlarged vascular bundles in node I in both the wild-type rice and *osfrdl1* mutant, but the mutant accumulated more Fe than the wild-type rice in this area. A stem-fed experiment with stable isotope ^57^Fe showed that the distribution of Fe in the anther and panicle decreased in the knockout line, but that in the flag leaf it increased compared with the wild-type rice. Taken together, our results show that *OsFRDL1* expressed in the upper nodes is required for the distribution of Fe in the panicles through solubilizing Fe deposited in the apoplastic part of nodes in rice.

## Highlight

*OsFRDL1* expressed in the upper nodes is required for the distribution of Fe to the panicles through solubilizing Fe deposited in the apoplastic part of nodes in rice.

## Introduction

Iron (Fe) is an essential element for plant growth and development because it is involved in the transport of electron in many metabolic processes such as respiration and photosynthesis, and is required as a cofactor of numerous enzymes (Hell and Stephan, 2003). The requirement for Fe for normal growth is between 50mg kg^−1^ and 150mg kg^−1^ in plant shoot dry weight depending on the plant species (Marschner, 1995), but different tissues also have different Fe requirements; for example, shoot apices require more Fe than other tissues for their active growth. Therefore, Fe in soil must be first taken up by the roots, followed by translocation from the roots to the shoots, and finally distributed to different tissues depending on their requirement. These processes are thought to be mediated by different transporters (Thomine and Vert, 2013), but only a few of them have been identified.

In dicots, Fe uptake is mediated by IRT1 (IRON REGULATED TRANSPORTER 1), a transporter for ferrous iron (Fe^2+^) (Eide *et al.*, 1996; Vert *et al.*, 2003). IRT1 is localized at the root epidermal cells and its expression is induced by Fe deficiency. In contrast, in gramineous plants, Fe is taken up in the form of an Fe^3+^–phytosiderophore complex, which is mediated by a transporter belonging to the YELLOW STRIPE/YELLOW STRIPE1-LIKE (YSL) family. The expression of *YS1* in maize, *HvYS1* in barley, and *OsYSL15* in rice is also induced by Fe deficiency (Curie *et al.*, 2001; Murata *et al.*, 2006; Inoue *et al.*, 2009). Furthermore, HvYS1 is also localized at the epidermal cells of barley roots (Murata *et al.*, 2006). Rice seems to have uptake systems mediated by both OsIRT1 and OsYSL15 (Ishimaru *et al.*, 2006; Inoue *et al.*, 2009), although their contribution to the uptake differs with soil conditions. It is speculated that in upland soil, OsYSL15-mediated uptake is predominant, while OsIRT1 plays a major role in Fe uptake in paddy soil, where ferrous Fe is rich. In addition, OsNramp5 (natural resistance-associated macrophage protein 5) and OsNramp1 also show transport activity for Fe^2+^, although their contribution to total Fe uptake is relatively small (Takahashi *et al.*, 2011; Ishimaru *et al.*, 2012; Sasaki *et al.*, 2012).

After the uptake, in Arabidopsis, part of Fe will be sequestered into the vacuoles through VIT1 (vacuole iron transporter 1) (Kim *et al.*, 2006) and IREG2/FPN2 (IRON REGULATED PROTEIN 2/ferroportin 2) (Schaaf *et al.*, 2006; Morrissey *et al.*, 2009) localized at the tonoplast of epidermal cells, while another part of Fe is loaded to the xylem by AtIREG1/FPN1, a plasma membrane-localized transporter at the pericycle cells (Morrissey *et al.*, 2009). In the same cells, *AtNramp3* and *AtNramp4* are also expressed, which pump Fe out of the vacuoles in Arabidopsis (Thomine *et al.*, 2003; Lanquar *et al.*, 2005). *OsVIT1* and *OsVIT2* in rice are also implicated in vacuolar sequestration of Fe (Zhang *et al.*, 2012).

Several studies have shown that a citrate efflux transporter belonging to the multidrug and toxic compound extrusion (MATE) family is required for the efficient translocation of Fe from the roots to the shoots. AtFRD3 (FERRIC REDUCTASE DEFECTIVE 3) in Arabidopsis, OsFRDL1 in rice, GmFRD3 in soybean, and HvAACT1 (Aluminum Activated Citrate Transporter 1) in barley are localized at the root pericycle cells and are responsible for releasing citrate to the xylem (Durrett *et al.*, 2007; Rogers *et al.*, 2009; Yokosho *et al.*, 2009; Fujii *et al.*, 2012). Knockout of these genes results in precipitation of Fe in the root stele and leaf chlorosis in Arabidopsis and rice (Durrett *et al.*, 2007; Yokosho *et al.*, 2009). In addition to citrate, several other chelators are also reported to be involved in internal mobilization of Fe in plants, including nicotianamine, deoxymugineic acids, and phenolic compounds. Release of phenolic acid to the xylem is mediated by PEZ1 (phenolics efflux zero 1) (Ishimaru *et al.*, 2011), while deoxymugineic acid is transported by TOM2 (transporter of mugineic acid 2) (Nozoye *et al.*, 2015).

Iron loaded to the xylem will be distributed to different organs and tissues depending on their demand, but the molecular mechanism underlying Fe distribution is poorly understood. Recently, in rice, it has been shown that nodes play an important role in the distribution of mineral elements (Yamaji and Ma, 2014). Several transporters required for the intervascular transfer at the nodes from the enlarged vascular bundle to the diffuse vascular bundle have been identified. In particular, transporters at the uppermost node I at the reproductive stage are most important for delivering mineral elements to the seeds. For example, three Si transporters (Lsi2, Lsi3, and Lsi6) localized in the different cell layers of node I are involved in the preferential distribution of Si to the panicle in rice (Yamaji *et al.*, 2015). Knockout of a node-localized Mn transporter, OsNramp3, also resulted in decreased distribution of Mn to the grains in rice (Yamaji *et al.*, 2013). By analyzing transcripts highly expressed in node I of rice (Yamaji *et al.*, 2013, 2015), we found that *OsFRDL1* was also highly expressed in this organ in addition to its expression in the roots. In the present study, we investigated the role of *OsFRDL1* expressed in the nodes in Fe distribution at the reproductive stage. We found that OsFRDL1 is required for the distribution of Fe to the grains through solubilizing apoplastic Fe in rice.

## Materials and methods

### Plant materials and growth condition in a paddy field

A *Tos-17* insertion line (*osfrdl1*) identified previously (Yokosho *et al.*, 2009), *pOsFRDL1* (*promoter of OsFRDL1*)-*GFP* (*green fluorescent protein*) transgenic rice, and its wild type (*Oryza sativa*, cv. Nipponbare) were used in this study. Seeds were germinated in tap water for 2 d in the dark at 30 ºC and then transferred to a net floating on a 0.5mM CaCl_2_ solution in a 1.2 liter pot. After 7 d, the seedlings were cultured in a nutrient solution (1/2 strength Kimura B solution) containing macronutrients (NH_4_)_2_SO_4_ (0.18mM), MgSO_4_·7H_2_O (0.27mM), KNO_3_ (0.09mM), Ca(NO_3_)_2_·4H_2_O (0.18mM), and KH_2_PO_4_ (0.09mM), and micronutrients MnCl_2_·4H_2_O (0.5 µM), H_3_BO_3_ (3 µM), (NH_4_)_6_Mo_7_O_24_·4H_2_O (1 µM), ZnSO_4_·7H_2_O (0.4 µM), CuSO_4_·5H_2_O (0.2 µM), and Fe-EDTA (20 µM). The pH of this solution was adjusted to 5.6 and the nutrient solution was renewed every 2 d.

Seedlings (3 weeks old) of both the wild type and *osfrdl1* were transplanted in a paddy field at the experimental farm of Okayama University in mid June, 2013. Each plot (0.7×0.7 m) contained eight seedlings, and four replicates were made for each line. At the end of October, plants were harvested and used for investigation of yield components and mineral determination.

### Determination of yield components

At harvest, the panicle number per plant was recorded. After air-drying, the spikelet number per panicle was counted from 10 panicles in each line. The percentage of filled spikelets (fertility) was determined using ~300 spikelets in a salt solution with a specific gravity of 1.06 (number of sinking spikelets/total number of spikelets×100). After air-drying, the straws and the filled spikelets were weighed and the 1000-grain weight of filled grains was calculated according to Tamai and Ma (2008). Total filled grain weight per plant (grain yield per plant) was calculated from panicle number×grain number per panicle×fertility (%)×grain weight (g).

### Determination of metal concentration in different organs

Harvested plants were separated into different organs including brown rice, husk, rachis, flag leaf blade, flag leaf sheath, node I, and the remaining part of the shoots (straw). Samples were dried at 70 ºC in an oven for several days and weighed. Digestion of samples was performed with concentrated nitric acid [60% (w/v)] at 140 ºC. The metal concentrations in the digestion solution were determined by inductively coupled plasma mass spectrometry (ICP-MS) (7700; Agilent, http://www.agilent.com/).

### Perls’ blue staining

Perls’ blue staining was performed with node I according to Yokosho *et al.* (2009). Node I of both wild-type rice and *osfrdl1* cultured in paddy fields was excised at the flowering stage. The cross-sections (100 μm) were sliced with LinearSlicer PRO10 (Dosaka EM). Sections were placed on microscope slides, and incubated with equal amounts of solutions of 4% (v/v) HCl and 4% (w/v) potassium ferrocyanide at room temperature for 1h. The signal was observed under an optical microscope (CKX41 with CCD camera FX630; Olympus).

### Expression pattern of *OsFRDL1* at the reproductive growth stage

To investigate the expression of *OsFRDL1* in different organs at the reproductive stage, different organs were sampled from the plants grown in the paddy field as described above at the flowering stage. Total RNA was extracted using an RNeasy plant mini kit (Qiagen, Hilden, NRW, Germany) and then converted to cDNA after DNase I treatment using a SuperScript II kit (Thermo Fisher Scientific, Waltham, MA, USA) following the manufacturer’s instructions. The expression level of *OsFRDL1* was determined by Sso Fast EvaGreen Supermix (Bio-Rad, Hercules, CA, USA), and quantitative real-time PCR was performed on CFX384 (Bio-Rad) using primers: *OsFRDL1* (forward) 5′-TCACCAATGCTAAGGCCTGC-3′ and (reverse) 5′-AACCACGGAAAACACCCTG-3′; *Histone H3* (forward) 5′-AGTTTGGTCGCTCTCGATTTCG-3′ and (reverse) 5′-TCAACAAGTTGACCACGTCACG-3′; and *Actin* (forward) 5′-GACTCTGGTGATGGTGTCAGC-3′ and (reverse) 5′-GGCTGGAAGAGGACCTCAGG-3′. Both *Histone H3* and *Actin* were used as internal standards, and relative expression was calculated by the ddCt method using CFX Manager software (Bio-Rad).

### Cell specificity of *OsFRDL1* expression

To investigate the cell specificity of *OsFRDL1* expression, we generated transgenic rice carrying the *OsFRDL1* promoter fused with *GFP*. The 2kb promoter region of *OsFRDL1* (−2001 to −1bp from the transcriptional start site) was amplified by PCR using primers 5′-AGAGCTCCAATCTAAATCGTACTAGAGACTG-3′ and 5′- TGTCGACGCTTCTCTCGGTTTGCCGAT-3′ from Nipponbare genomic DNA. The fragment was then subcloned into the binary vector pPZP2H-sGFP (Fuse *et al.*, 2001) to generate *pOsFRDL1*-*GFP* using the cloning sites *Sac*I and *Sal*I. This construct was subsequently introduced into *Agrobacterium tumefaciens* (strain EHA101). Callus was induced from mature embryos of rice cultivar Nipponbare. *Agrobacterium*-mediated transformation was performed according to the protocol of Hiei *et al.* (1994)

Immunostaining with an antibody against GFP was performed with the transgenic plants according to Yamaji and Ma (2007). The transgenic line and wild type were grown in the nutrient solution as described above. At the flowering stage, the flag leaf blade and sheath, node I, peduncle (internode I), rachis, husk, and anther were sampled and subjected to immunostaining. The signal was observed with a confocal laser scanning microscope (LSM700; Carl Zeiss).

### Pollen grain viability staining

Pollen grains from both the wild type and *osfrdl1* grown in the paddy field as described above were sampled before flowering for viability testing and measurement of anther and pistil size before flowering. Three spikelets from one plant were randomly sampled from a total of four plants. Photographs of pollen were taken under an optical microscope (VHX2000; KEYENCE), and the size of the anther and pistil was measured from the image data. The pollen grains were stained with 1% KI–I_2_ solution for 5min at room temperature. Photographs were taken under an optical microscope (CKX41 with CCD camera FX630; Olympus). The pollen viability ratio was calculated as follows: pollen stained with purple color/total pollen in the screen×100. Four biological replicates were performed.

### Short-term stem-feeding experiment with ^57^Fe, rubidium, and strontium

Both the wild type and *osfrdl1* were cultivated in the paddy field as described above. At the flowering stage, the plants were cut at internode III (3cm from node II) with a razor blade. A 5 µM concentration of ^57^FeCl_3_ (96.1% ^57^Fe, TRACE Sciences International) with 1 µM rubidium (Rb) and 1 µM strontium (Sr) in 0.5mM CaCl_2_ was fed from the cut end in a growth chamber at 25 ºC, 80% relative humidity. After incubation for 24h, the flag leaf, node I, and panicle were harvested separately and dried at 70 ºC in an oven for several days. Concentrations of ^57^Fe, Rb, and Sr in each part were determined by ICP-MS using isotope mode after digestion as described above.

## Results

### Expression pattern of *OsFRDL1* at the flowering stage

**Fig. 1.**
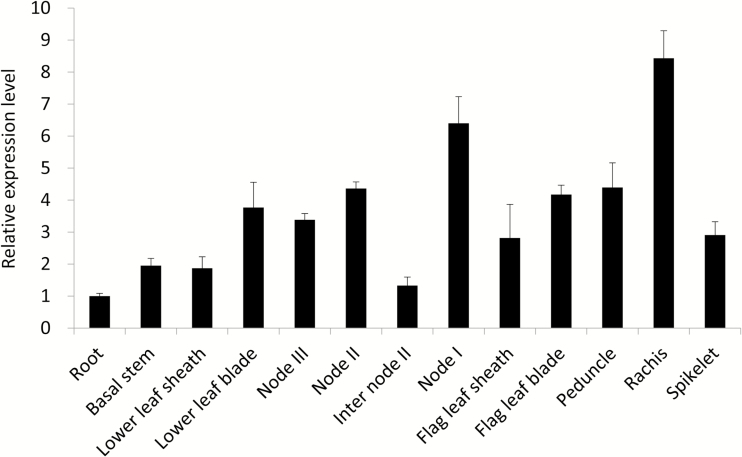
Expression of *OsFRDL1* in different organs at the flowering stage of rice. Different organs were sampled from rice (cv Nipponbare) cultivated in a paddy field at the flowering stage. The expression level was determined by quantitative RT–PCR. *Actin* and *Histone H3* were used as internal standards. The expression level relative to that in the roots is shown. Data are means ±SD of three biological replicates.

*OsFRDL1* is mainly expressed in the roots at the vegetative growth stage of rice (Inoue *et al.*, 2004; Yokosho *et al.*, 2009). To investigate the tissue specificity of *OsFRDL1* expression at the reproductive stage, we took different organs of rice grown in a paddy field at the flowering stage and determined their expression level by a quantitative reverse transcription–PCR (RT–PCR). At the flowering stage, in addition to its expression in the roots, higher expression of *OsFRDL1* was found in the above-ground parts including nodes, rachis, peduncle, spikelet, and leaves (Fig. 1). The expression levels of the rachis and uppermost node in particular were higher than that of other organs (Fig. 1).

**Fig. 2.**
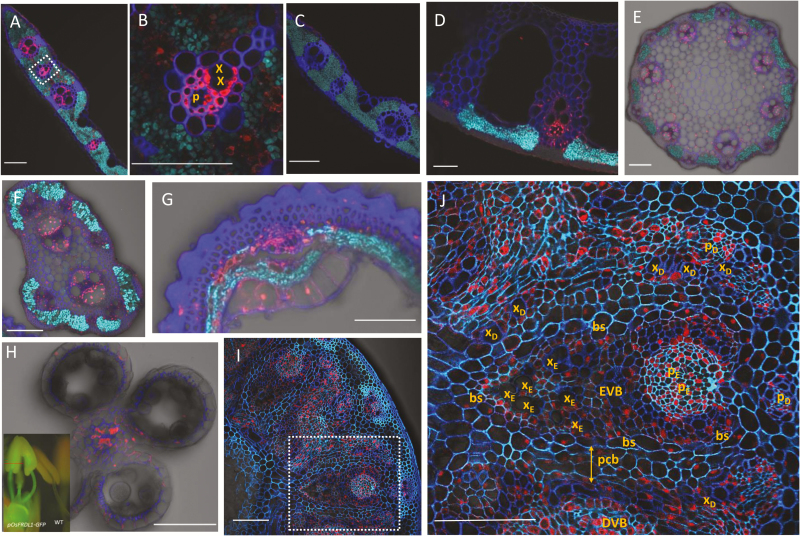
Cell specificity of *OsFRDL1* expression at the reproductive growth stage of rice. Immunostaining with a GFP antibody was performed in different tissues of transgenic plants carrying *pOsFRDL1-GFP*. (A–C) Flag leaf blade of transgenic rice (A, B) and wild-type rice (C). (D–J) Flag leaf sheath (D), peduncle (internode I) (E), rachis (F), husk (G), stamen (H), and node I (I, J) of the transgenic plants. The red line shows the cross-section position in (H). Scale bars=100 μm. x, xylem; p, phloem; x_E_, xylem of enlarged vascular bundle (EVB); p_E_, phloem of enlarged vascular bundle; x_D_, xylem of diffuse vascular bundle (DVB); p_D_, phloem of diffuse vascular bundle; bs, bundle sheath of enlarged vascular bundle; pcb; parenchyma cell bridge.

To examine the cell specificity of *OsFRDL1* expression, we generated transgenic rice carrying the *OsFRDL1* promoter (2 kb) fused with *GFP*. Immunostaining with an antibody against GFP showed that *OsFRDL1* promoter activity was detected at the vascular tissue of the leaf blade of the transgenic line (Fig. 2A, B). However, the signal was not detected in the leaf of wild-type rice (Fig. 2C), indicating the specificity of the antibody used. In addition to this specificity, we confirmed that the signal was detected in vascular tissue in the roots (data not shown), which is the same as previous results by immunostaining with an anti-OsFRDL1 antibody and by GUS (β-glucuronidase) staining in *OsFRDL1* promoter–GUS transgenic rice (Inoue *et al.*, 2004; Yokosho *et al.*, 2009). The GFP signal was also observed in the vascular tissues of the leaf sheath (Fig. 2D), peduncle (Fig. 2E), rachis (Fig. 2F), and husk (Fig. 2G). These results for the leaf blade and flower are consistent with the previously seen tissue- specific expression pattern (Inoue *et al.*, 2004). The expression of the *OsFRDL1* promoter was also observed in the vascular tissues of the filament but not in the anthers (Fig. 2H). In node I, the expression of the *OsFRDL1* promoter was detected in enlarged vascular bundles, diffuse vascular bundles, and the intervening parenchyma tissues (parenchyma cell bridges) (Fig. 2I, J).

### Knockout of *OsFRDL1* resulted in significant reduction of grain yield

**Fig. 3.**
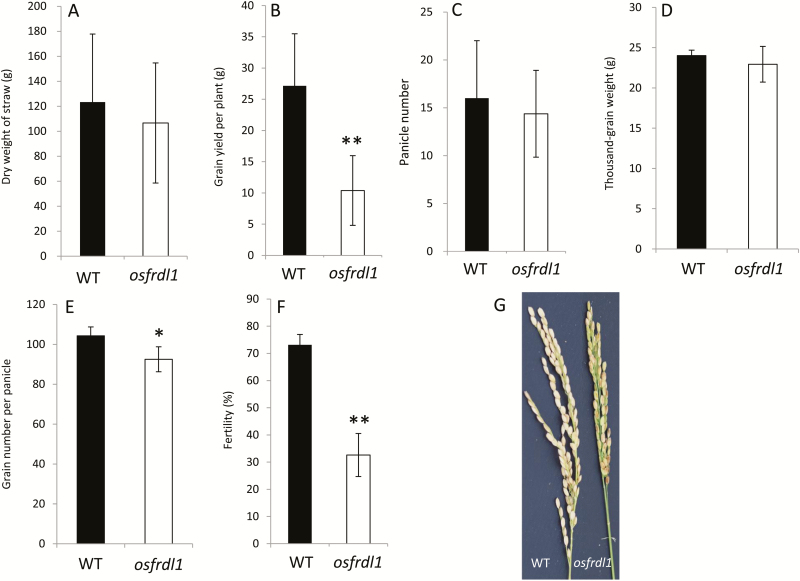
Phenotypic analysis of the *osfrdl1* mutant grown in a paddy field. Both the *osfrdl1* mutant and its wild-type (WT) rice (cv. Nipponbare) were cultivated in a paddy field until ripening. (A) Dry weight of straw, (B) grain yield per plant, (C) panicle number per plant, (D) 1000-grain weight of filled grains, (E) grain number per panicle, (F) percentage of filled spikelets. Error bars represent ±SD of eight biological replicates. (G) Panicles at harvest. Asterisks above the bars indicate significant differences (*P<0.05; **P<0.01) compared with the wild-type rice.

To investigate the role of *OsFRDL1* at the reproductive growth stage, we grew a *Tos-17* retrotransposon insertional line (*osfrdl1*) identified previously (Yokosho *et al.*, 2009) and its wild-type rice in a paddy field until ripening. A previous study showed that *osfrdl1* (ND8025) is a knockout line without expression of *OsFRDL1* (Yokosho *et al.*, 2009). There was no difference in the biomass of the straw between *osfrdl1* and the wild type (Fig. 3A). However, the grain yield was decreased by ~60% in *osfrdl1* compared with the wild type (Fig. 3B). Among yield components, there was no difference in the panicle number and 1000-grain weight of filled grains between *osfrdl1* and the wild type (Fig. 3C, D), but the grain number per panicle was slightly less in the mutant than in the wild type (Fig. 3E). The most significant difference between *osfrdl1* and the wild type was observed in the percentage of filled grains (fertility); the fertility of *osfrdl1* was decreased by ~55% compared with the wild type (Fig. 3F, G).

**Fig. 4.**
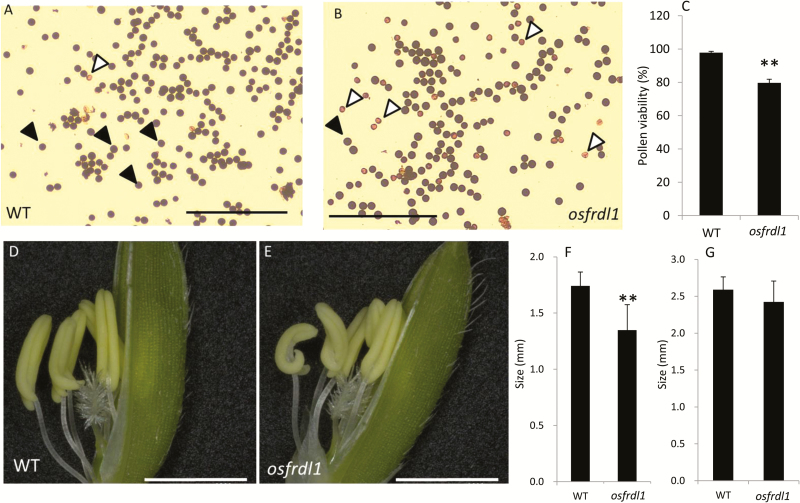
Pollen viability of wild-type (WT) rice and the *osfrdl1* mutant. Both the WT and *osfrdl1* mutant were grown in a paddy field until the flowering stage. Pollen grains were sampled and stained with 1% KI–I_2_ solution in the WT (A) and *osfrdl1* (B). Black arrowheads indicate normal pollen (purple color grains), while white arrowheads indicate abnormal pollen (weak red color grains). Black bars=500 μm. Percentage pollen viability (C) was calculated based on the different colors (A, B). Data are means ±SD of four biological replicates. Asterisks above the bars indicate significant differences (**P<0.01) compared with the WT rice. (D–G) Flower of the WT (D) and the *osfrdl1* mutant (E), and the size of the anther (F) and pistil (G). Data are means ±SD of 35 replicates for anther size and five for pistil size. Asterisks above the bars indicate significant differences (**P<0.01) compared with the wild-type rice. White bars=2mm.

To examine whether the reduced fertility results from pollen viability, we performed KI–I_2_ staining. The amount of normal pollen grains stained with purple color was more in the wild type than in the mutant (Fig. 4A, B), while more abnormal pollen grains stained with weak red color were observed in the mutant (Fig. 4B). As a result, the pollen viability was 20% lower in *osfrdl1* than in the wild type (Fig. 4C). Furthermore, the size of the anther was smaller in *osfrdl1* compared with the wild type, but there was no difference in the size of the pistil (Fig. 4D–G).

### Metal concentration in different organs of rice grown in a paddy field

**Fig. 5.**
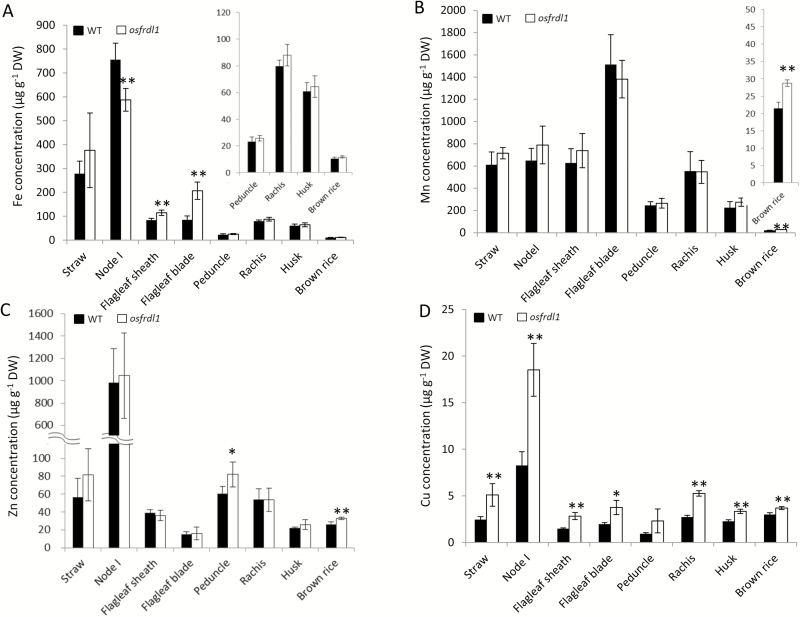
Metal concentration in different organs at the reproductive growth stage of rice. Both the wild type (WT) and the *osfrdl1* mutant were grown in a paddy field until ripening. After harvest, different organs were separated and subjected to determination of Fe (A), Mn (B), Zn (C), and Cu (D). Error bars represent ±SD of four biological replicates. Asterisks above the bars indicate significant differences (*P<0.05; ** *P*<0.01) compared with the WT rice.

The concentration of Fe and other metals in different organs was compared between the wild type and *osfrdl1* grown in a paddy field. A higher Fe concentration in the flag leaf sheath and blade was found in *osfrdl1* than in the wild type (Fig. 5A), while in node I, the Fe concentration was lower in the mutant than in the wild type. There was no difference in the Fe concentration in other organs including remaining straw, peduncle, rachis, husk, and brown rice in the two lines (Fig. 5A). No difference was found in the concentration of Mn and Zn in all organs except brown rice for Zn and Mn, and peduncle for Zn between the wild type and *osfrdl1* (Fig. 5B, C). However, the Cu concentration in all organs was higher in the mutant than in the wild type (Fig. 5D).

### Fe deposition staining of node I

**Fig. 6.**
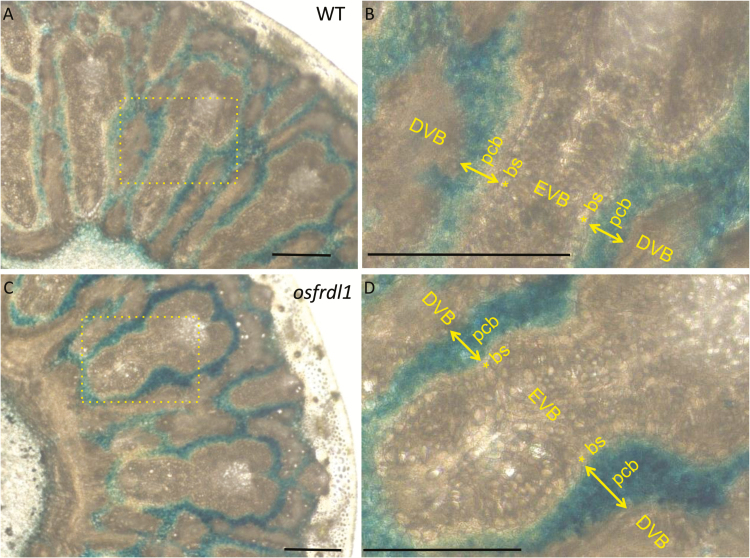
Iron deposition in node I with Perls’ blue staining. Both the wild type (WT; A, B) and the *osfrdl1* (C, D) mutant were grown in a paddy field until the flowering stage. Node I was sliced and subjected to Perls’ blue staining. Blue color shows Fe deposition. Scale bars=100 μm. EVB, enlarged vascular bundle; DVB, diffuse vascular bundle; bs, bundle sheath of enlarged vascular bundle; pcb, parenchyma cell bridge.

We examined Fe deposition in node I of both *osfrdl1* and the wild type by Perls’ blue staining. In both lines, Fe was deposited in parenchyma cell bridges; a few cell layers next to the apoplastic barrier at the bundle sheath of enlarged vascular bundles (Fig. 6). This deposition was located between the enlarged vascular bundle and diffuse vascular bundle (Fig. 6). The signal of Fe deposition at these cell layers was stronger in *osfrdl1* than in the wild type (Fig. 6).

### Knockout of *OsFRDL1* altered Fe distribution at the flowering stage

**Fig. 7.**
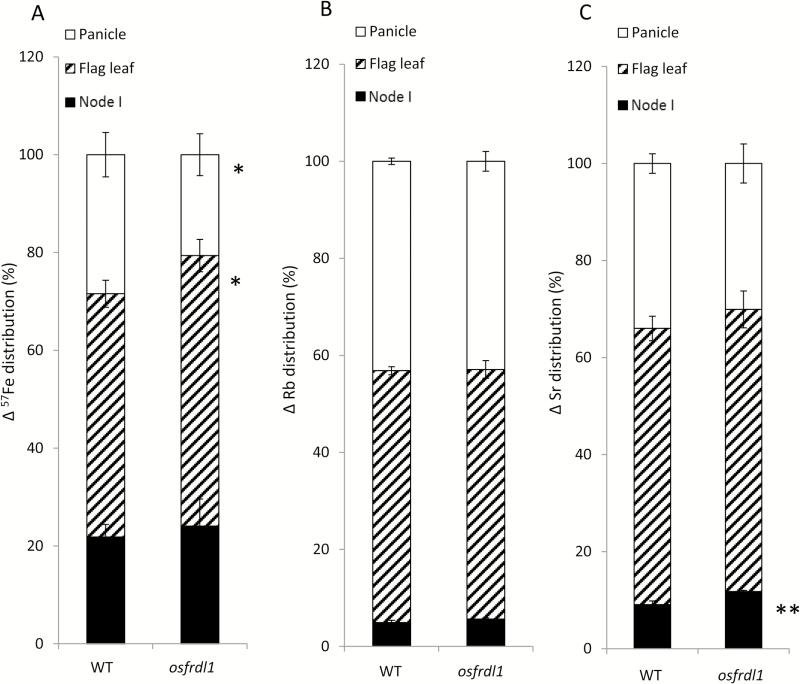
Distribution of ∆^57^Fe, rubidium (Rb), and strontium (Sr) to different organs at the reproductive growth stage. Both the wild type (WT) and *osfrdl1* were cultivated in a paddy field until the flowering stage. The plants were cut at internode III below node II and fed with 5 µM ^57^FeCl_3_, 1 µM Rb, and 1 µM Sr in a 0.5mM CaCl_2_ solution. After 24h, the flag leaf, node I, and panicle were separately harvested and subjected to determination of ^57^Fe, Rb, and Sr. The distribution ratios of Δ^57^Fe (A), Rb (B), and Sr (C) in different organs were calculated. Error bars represent ±SD of three biological replicates. Asterisks above the bars indicate significant differences (*P<0.05; **P<0.01) compared with the WT rice.

OsFRDL1 is involved in the translocation of Fe from the roots to the shoots (Yokosho *et al.*, 2009). To exclude its role in the distribution of Fe, we performed a short-term stem-feeding experiment with the ^57^Fe stable isotope at the flowering stage. Ferric ^57^Fe was fed to the cut end of internode III (3cm from node II) with Rb and strontium Sr as markers of phloem and xylem transport, respectively (Kuppelwieser and Feller, 1991). The results showed that the distribution of newly absorbed ^57^Fe (∆^57^Fe) to the flag leaf was increased, but that to the panicles was decreased in *osfrdl1* compared with the wild type (Fig. 7A). Although the Fe staining intensity in *osfrdl1* was stronger than that in the wild type (Fig. 6), there was no difference in ^57^Fe distribution of the node between the wild type and *osfrdl1* (Fig. 7A). This difference could be attributed to short exposure (24h) to ^57^Fe. On the other hand, there was no difference in the distribution of Rb and Sr to the flag leaf and panicles between the two lines (Fig. 7B, C). Taken together, these results indicate that OsFRDL1 in node I plays an important role in the distribution of Fe at the reproductive growth stage.

## Discussion

OsFRDL1 is a plasma membrane-localized efflux transporter for citrate (Inoue *et al.*, 2004; Yokosho *et al.*, 2009). At the vegetative growth stage of rice, *OsFRDL1* is mainly expressed in the pericycle cells of mature root regions and mediates release of citrate to the xylem, which is required for the translocation of Fe from the roots to the shoots (Inoue *et al.*, 2004; Yokosho *et al.*, 2009). In the present study, we found that *OsFRDL1* is also involved in the distribution of Fe to panicles at the reproductive growth stage. *OsFRDL1* showed higher expression in node I and other reproductive organs (Fig. 1). Knockout of this gene resulted in increased Fe deposition in node I and decreased distribution of Fe to the panicles (Figs 5–7). These alterations may be associated with decreased pollen viability and grain fertility (Figs 3, 4).

**Fig. 8.**
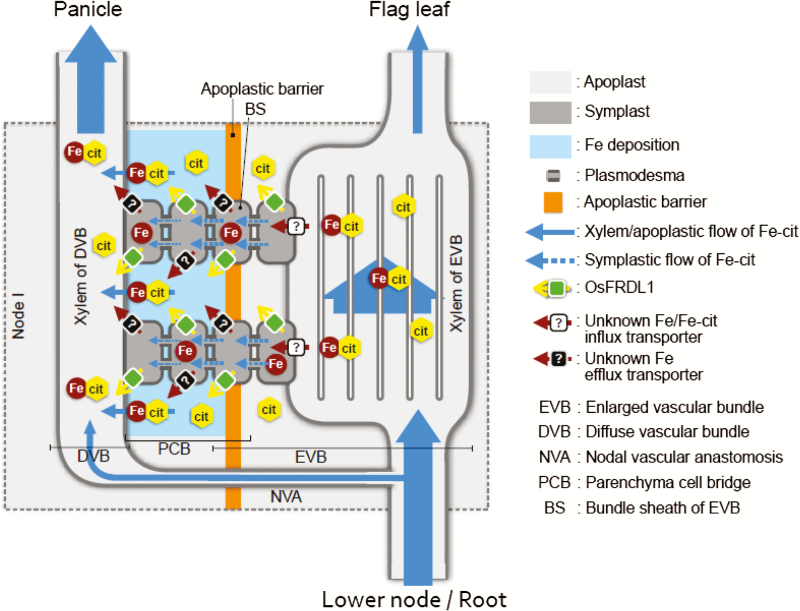
Schematic presentation of the role of OsFRDL1 in node I. The Fe–citrate complex in the xylem of enlarged vascular bundle in node I is unloaded by an unidentified influx transporter to the xylem parenchyma cells. Fe is then symplastically transported to the bundle sheath cells, where an apoplastic barrier is located. Part of Fe is released from the bundle sheath cells and/or parenchyma cell bridge through an unidentified efflux transporter and deposited in the apoplast of the parenchyma cell bridge as an Fe pool. OsFRDL1 localized at most cells of node I mediates release of citrate from the cells, which solubilizes apoplastic Fe deposited at the parenchyma cell bridge. The Fe–citrate complex is finally re-loaded to the diffuse vascular bundle for subsequent loading of Fe to the panicle.

Iron plays essential roles in pollen development (Lanquar *et al.*, 2005; Roschzttardtz *et al.*, 2011; Schuler *et al.*, 2012). To deliver Fe to the pollen and other reproductive organs efficiently in gramineous plants, an intervascular transfer of Fe from the enlarged vascular bundles to the diffuse vascular bundles is required in the nodes (Fig. 8; Yamaji and Ma, 2014). However, transporters involved in this process have not been identified. Iron in the xylem could form complexes with citrate (Tiffin, 1970; Durrett *et al.*, 2007; Yokosho *et al.*, 2009; Rellán-Alvarez *et al.*, 2010), phytosiderophore (Kakei *et al.*, 2009; Nozoye *et al.*, 2015), and phenolic compounds (Ishimaru *et al.*, 2011), but the Fe–citrate complex is the dominant form because the citrate concentration in the xylem is much higher than that of other chelators. For preferential distribution of Fe to the reproductive organs through intervascular transfer, the Fe–citrate complex transported to the upper nodes through an enlarged vascular bundle must be unloaded first to the xylem parenchyma cells and then loaded to the xylem of the diffuse vascular bundle (Fig. 8). In the *osfrdl1* mutant, due to decreased citrate in the xylem, less Fe–citrate will be unloaded from the xylem. As a result, more unchelated Fe will be transported to the flag leaf following the transpiration, resulting in higher Fe in the flag leaf of the mutant. After Fe–citrate is loaded from the xylem, citrate is again required for mobilization of Fe between two vascular bundles; therefore, knockout of *OsFRDL1* resulted in more Fe precipitation in the parenchymal cell bridge.

Recently, an apoplastic barrier was found to be located on the bundle sheath of the enlarged vascular bundles (Yamaji *et al.*, 2015). Therefore, Fe in the xylem parenchyma cells must thus be symplastically transported to the bundle sheath cells (Fig. 8). We found that Fe was heavily deposited in the parenchyma cell bridges next to this apoplastic barrier (Fig. 6), indicating that part of Fe is transported out again from the bundle sheath cells and/or parenchyma cell bridges to the apoplast. This Fe deposition seems to be an Fe pool, which is necessary to avoid excess Fe concentration in the symplast (Curie and Briat, 2003). Therefore, to utilize this Fe for subsequent transport of Fe to the panicles in rice, a solubilization process by suitable chelating molecules is required. *OsFRDL1* expressed in node I seems to be involved in this solubilization process by releasing citrate to the apoplastic space. This is supported by the finding that more Fe was deposited in the parenchyma cell bridge of node I in *osfrdl1* (Fig. 6). Furthermore, knockout of *OsFRDL1* resulted in decreased distribution of Fe to the panicles, but increased distribution to the flag leaf and higher Fe concentration in the flag leaf (Figs 5, 7). This role of OsFRDL1 in rice is similar to that of AtFRD3 in Arabidopsis. In addition to its role in root to shoot translocation of Fe, a recent study showed that *AtFRD3* also plays an important role in proper Fe transport to the embryo and pollen (Roschzttardtz *et al.*, 2011). *AtFRD3* is expressed in the seeds and flowers of Arabidopsis, and knockout of this gene results in a defect of early germination and almost complete sterility (Roschzttardtz *et al.*, 2011). In addition to the expression of *OsFRDL1* in the nodes, it was also expressed in the vascular tissues of other organs including the leaf, peduncle, rachis, husk, and filament (Fig. 2). Similar to its role in the nodes, OsFRDL1 in these tissues is probably also involved in the solubilization of apoplastic Fe. These findings suggest that both AtFRD3 in Arabidopsis and OsFRDL1 in rice have similar role in solubilizing apoplastic Fe in reproductive organs.

Under Fe-limited conditions, knockout of *OsFRDL1* resulted in a significant decrease of Fe concentration in the shoots (Yokosho *et al.*, 2009). However, no difference was found in Fe concentration of straw between the wild type and *osfrdl1* when grown in a paddy field (Fig. 5). This inconsistency may be attributed to the Fe concentration in the hydroponic culture solution or soil solution. In paddy fields, ferrous Fe in soil solution is very high due to soil reduction, resulting in higher Fe accumulation in the mutant shoot. In fact, when the *osfrdl1* mutant was grown hydroponically in a high Fe concentration, the Fe concentration of the shoots also significantly increased (Yokosho *et al.*, 2009). Therefore, the reduced pollen viability and grain fertility in the mutant is not due to the decreased root to shoot translocation of Fe, but to the incorrect distribution of Fe to different organs at the flowering stage. Among other essential metals, there was almost no difference in the accumulation of Zn and Mn between the wild type and *osfrdl1* mutant (Fig. 5), but the Cu concentration in all organs was higher in the mutant than in the wild type. The reason for this increased Cu accumulation in the mutant shoots is unknown. One possibility is that internal Fe deficiency in the mutant due to incorrect Fe distribution at the reproductive stage may induce expression of some unknown Cu uptake transporter genes. Studies showed that there was crosstalk between Fe and Cu in plants (Bernal *et al.*, 2012; Perea-García *et al.*, 2013; Waters *et al.*, 2014). In rice, the expression of *OsYSL16*, which is a Cu-nicotianamine transporter gene responsible for delivering Cu from the old tissues to the young tissues through phloem transport, was up-regulated by Fe deficiency in the roots (Zheng *et al.*, 2012). The expression of *OsCOPT* (*COPPER TRANSPORTER*) *2*, *5*, and *7* in the shoots was also induced by Fe deficiency although their exact role remains to be investigated (Yuan *et al.*, 2011). In Arabidopsis, knockout of *AtFRD3* resulted in increased accumulation of Mn and Co (Roschzttardtz *et al.*, 2011).

In conclusion, in addition to its role in translocation of Fe from the roots to shoots, OsFRDL1 also plays an important role in distribution of Fe to the reproductive organs by releasing citrate to solubilize apoplastic Fe in the vascular tissues of rice.

## Abbreviations:

AACT1: Aluminum Activated Citrate Transporter 1
COPT: COPPER TRANSPORTER
FRD3: FERRIC REDUCTASE DEFECTIVE 3
FRDL1: FERRIC REDUCTASE DEFECTIVE LIKE 1
FPN: ferroportin
GFP: green fluorescent protein
IRT1: IRON REGULATED TRANSPORTER 1
IREG: IRON REGULATED PROTEIN
Lsi1: Low silicon rice 1
MATE: multidrug and toxic compound extrusion
Nramp: natural resistance-associated macrophage protein
pOsFRDL1: promoter of OsFRDL1
PEZ1: Phenolics efflux zero 1
TOM: transporter of mugineic acid
VIT: vacuole iron transporter
YSL: YELLOW STRIPE1 LIKE.
